# Occlusion pressure and blood pressure adaptations following low-load blood flow restriction training versus moderate-load training: a pilot randomized clinical trial in patients with knee osteoarthritis

**DOI:** 10.3389/fphys.2025.1693341

**Published:** 2026-01-20

**Authors:** Mikhail Santos Cerqueira, Rafael Pereira, Daniel Germano Maciel, Cláudia Thais Pereira Pinto, Nicholas Rolnick, Wouber Hérickson de Brito Vieira

**Affiliations:** 1 Neuromuscular Performance Analysis Laboratory, Department of Physical Therapy, Federal University of Rio Grande do Norte, Natal, Rio Grande do Norte, Brazil; 2 Postgraduate Program in Physical Education, State University of Southwest Bahia, Jequié, Bahia, Brazil; 3 Integrative Physiology Research Center, Department of Biological Sciences, State University of Southwest Bahia, Jequié, Bahia, Brazil; 4 Department of Physical Therapy, Federal University of Paraíba, João Pessoa, Paraíba, Brazil; 5 The BFR PROS, New York, NY, United States

**Keywords:** vascular occlusion exercise, KAATSU training, arterial hypertension, pain, resistance training

## Abstract

**Objective:**

This pilot study investigated potential adaptations in limb occlusion pressure (LOP) and compared LOP between low-load resistance training with blood flow restriction (LL-BFR) and traditional moderate-load training (ML) over 9 weeks in patients with knee osteoarthritis (KOA). Secondarily, we compared systolic blood pressure (SBP) and diastolic blood pressure (DBP) adaptations between these groups.

**Methods:**

Seventeen KOA patients were randomly assigned to the LL-BFR or ML groups. The LL-BFR group performed 75 repetitions (10% 1RM; 60% of LOP). The ML group performed 24 repetitions (60% 1RM; 10% [SHAM] of LOP). In both groups, exercises involving LOP were bilateral hack machine squat and knee extension exercises. LOP, SBP, and DBP were measured before and every 3 weeks until the ninth training week.

**Results:**

After 9 weeks, there were no statistically significant changes in LOP, SBP, or DBP within or between groups. LOP decreased by −32.9 mmHg (95% CI: −68.9 to 3.2) in LL-BFR and −17.2 mmHg (95% CI: −49.0 to 14.6) in ML, achieving clinical significance [relative error variance (REV) = 14.6 mmHg]. SBP decreased by −7.5 mmHg (95% CI: −15.6 to 0.6) in LL-BFR and −1.1 mmHg (95% CI: −8.2 to 6.0) in ML. DBP decreased by −3.7 mmHg (95% CI: −9.2 to 1.7) in LL-BFR and −1.9 mmHg (95% CI: −6.8 to 2.9) in ML.

**Conclusion:**

We observed a non-significant trend toward a reduction in LOP following 9 weeks of LL-BFR in patients with KOA, with a mean point estimate that exceeded a pre-defined threshold for clinical significance, although the wide confidence intervals indicate substantial uncertainty. Furthermore, SBP and DBP showed no significant changes, and no group differences emerged across outcomes. Practically, these findings suggest that LOP remains stable throughout a rehabilitation program, potentially reducing the burden of frequent LOP reassessment in clinical LL-BFR applications.

**Trial registration:**

https://ensaiosclinicos.gov.br/rg/RBR-6pcrfm/.

## Introduction

Knee osteoarthritis (KOA) is among the most prevalent musculoskeletal disorders, with an estimated global prevalence of 364.6 million cases (Yang et al., 2023). This chronic condition is strongly associated with disability, joint pain, stiffness, and impaired function (Jacobs et al., 2024; Li et al., 2024). Projections indicate that KOA prevalence will increase by approximately 75% by 2050, driven by global population growth and an aging demographic (Steinmetz et al., 2023). Such growth is expected to intensify the personal, social, and economic burden of the disease.

Quadriceps weakness is a recognized risk factor for both the onset and progression of KOA. It is closely associated with greater pain severity, reduced mobility, and functional limitations (Øiestad et al., 2022; Shen et al., 2024). Consequently, interventions targeting quadriceps strength are critical for mitigating symptoms and slowing disease progression in patients with KOA.

Resistance exercise is considered a first-line treatment for KOA (Gibbs et al., 2023). However, many patients with joint pain are unable to tolerate traditional moderate-load training (Cerqueira and Brito Vieira, 2019). In this context, low-load resistance exercise combined with blood flow restriction (BFR) has emerged as a widely applied strategy for quadriceps strengthening in KOA patients (Jacobs et al., 2025; Ogrezeanu et al., 2025; Sørensen et al., 2025). BFR involves the application of a pneumatic cuff placed proximally on the exercising limb, exerting a specific limb occlusion pressure (LOP) that partially restricts arterial inflow and fully restricts venous outflow from the working muscle (Loenneke et al., 2025; Souza et al., 2025). Accurate determination and prescription of individualized LOP are essential to ensure that all patients receive a consistent training stimulus while minimizing the risk of adverse events (Spranger et al., 2015; Rolnick et al., 2021).

Several techniques have been proposed for determining LOP, including Doppler ultrasound (Bezerra et al., 2017), handheld Doppler (Laurentino et al., 2018), pulse oximetry (Zeng et al., 2019), and predictive equations (Hunt et al., 2016). Despite these methods, no consensus exists on the justification for specific LOP prescriptions (Clarkson et al., 2020), nor on whether LOP should be adjusted during long-term (≥4 weeks) BFR training interventions (Cerqueira et al., 2021a). Some have recommended measuring LOP before each training session (Ingram et al., 2017), yet this practice may be burdensome in clinical contexts (Mattocks et al., 2019).

Low-load exercise protocols using fixed percentages of LOP (e.g., 40% or 80%) maintained throughout rehabilitation have been proposed for KOA (Wang et al., 2022). However, there is little knowledge about the need for adjustment in LOP throughout training sessions (Loenneke et al., 2025). In young, healthy populations, LOP appears stable across interventions (Mattocks et al., 2019), but this question has not been adequately investigated in clinical populations such as KOA patients (Cerqueira et al., 2021a). Furthermore, although low-load blood flow restriction (LL-BFR) has been shown to acutely elevate systolic blood pressure (SBP) and diastolic blood pressure (DBP) in older adults (Zhang et al., 2022), no studies to date have examined these parameters longitudinally in KOA.

Thus, the primary objective of this study was to investigate potential adaptations in LOP and compare LOP responses between LL-BFR training and traditional moderate-load training over 9 weeks in patients with KOA. The secondary objective was to evaluate SBP and DBP adaptations between the two groups.

## Materials and methods

### Study design

This study was designed as a randomized, sham-controlled clinical trial with allocation concealment, blinded assessors, and blinded volunteers. The protocol was approved by the Research Ethics Committee of the Federal University of Rio Grande do Norte (CAAE: 91753618.4.0000.5537) and prospectively registered in the Brazilian Clinical Trials Registry (RBR-6pcrfm). All participants provided written informed consent prior to enrollment.

Eligible participants with KOA were randomized to either the LL-BFR group or traditional moderate-load exercise (ML) groups. Randomization was performed using www.randomization.com, with balanced block permutations stratified by the presence of unilateral or bilateral KOA. Allocation was concealed in sequentially numbered, sealed, opaque envelopes, which were prepared in advance by an independent assistant not otherwise involved in the study.

### Participants

Participants aged 50 years or older with a clinical diagnosis of KOA were included. The inclusion criteria were as follows: 1. postmenopausal status (for women); 2. height between 1.50 and 1.75 m; 3. body mass index (BMI) between 18.5 and 35 kg∙m^−2^; 4. Lequesne Questionnaire score between 5 and 13 (moderate to very severe KOA); 5. absence of diabetes; 6. absence of chronic, uncontrolled arterial hypertension; 7. absence of peripheral vascular or cerebrovascular diseases and no history of exercise-limiting cancer; 8. no other orthopedic/neurological diseases affecting gait and no systemic inflammatory myoarticular disorder; 9. no knee surgery within the past 6 months; 10. no physiotherapy treatment or lower-limb strengthening program within the past 3 months; and 11. no regular lower-limb physical activity (two or more times a week), except for walking. Participants continued any medications they had been taking regularly for at least 3 months prior to study initiation. Exclusion criteria were 1. withdrawal of consent and 2. initiation of a regular exercise program after starting the intervention.

To characterize the sample, self-reported worst knee pain over the past week was assessed using a visual analog scale (VAS, 0–10 cm), and KOA severity was evaluated with the Lequesne Questionnaire.

### Limb occlusion pressure

**FIGURE 1:**
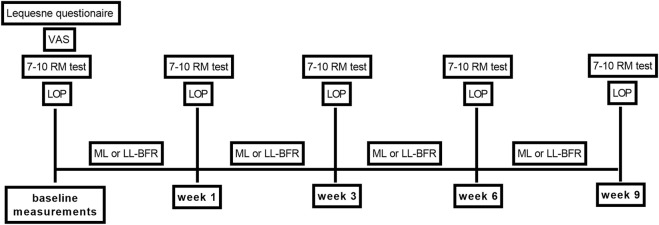
Study design. VAS, visual analogic scale; RM, repetition maximum; LOP, limb occlusion pressure; LL-BFR, low-load exercise with blood flow restriction; ML, traditional moderate-load exercise.

LOP was determined using a handheld Doppler device (DV 2001, MEDPEJ®, Ribeirão Preto, Brazil) following previously described protocols (Bezerra et al., 2017; Cerqueira et al., 2021a). In brief, a Doppler transducer (5–10 MHz) was positioned to detect the auscultatory signal of the posterior tibial artery while a pneumatic cuff was inflated. The same cuff configuration was used for both LOP assessment and during BFR exercise sessions. The design and methodology for BFR application are presented in Chart 1. LOP and estimated one-repetition maximum (1RM) were reassessed and adjusted every 3 weeks (Figure 1).

**Table:** CHART 1 Apparatus design and methodology for blood flow restriction application.

Manufacturer and model	Custom-made, manually inflatable pneumatic cuff (Premium®, Duque de Caxias, Brazil); fluctuations in prescribed pressure were monitored and regulated during the interset interval
Method of pressure measurement	Manual device (Doppler ultrasound); incremental pressure protocol. Adapted from Bezerra et al. (2017)
Pressure regulation	Unregulated
Cuff width	10 cm wide × 80 cm long
Material	Nylon
Type of the internal bladder system	Single-chambered
Cuff shape	Straight
Internal bladder length	The cuff was positioned in the subinguinal region with the cuff bladder enveloping the entire medial portion of the thigh and part of the anterior and posterior portions of the thigh
Limb occlusion pressure	See Table 2
Posture used for measurement of limb occlusion pressure	Supine posture; volunteer was instructed to remain relaxed and still during the procedure
Timing of pressure application	Continuous pressure was applied immediately before exercises and maintained during the sets and interval between sets. The cuff was deflated during the 2-min rest period between exercises (hack machine squats and knee extensions)

Adapted from Hughes et al. (2025).

### Exercise protocols

Participants attended two supervised training sessions per week for 9 weeks, led by a trained physiotherapist. Each session began with a 5-min self-regulated light-intensity warm-up on a stationary bicycle. Both groups then performed bilateral thigh muscle strengthening exercises, consisting of hack machine squats (0°–60° knee flexion) and knee extensions (90°–45° of knee flexion) (Giles et al., 2017; Ferraz et al., 2018). In addition, participants completed strengthening exercises for trunk, hip, and calf muscles (Cerqueira and Brito Vieira, 2019). 1RM was estimated using the Brzycki equation (McNair et al., 2011; Cook et al., 2017), based on the maximum load achieved during a 7–10 RM test. Training loads were reassessed and adjusted every 3 weeks.

The LL-BFR group performed one set of 30 repetitions, followed by three sets of 15 repetitions at 10% of the estimated 1 RM, with 30 s of rest between sets, using the hack machine squat and knee extension exercises. These exercises were performed with 60% of LOP applied bilaterally. The ML group performed three sets of eight repetitions at 60% of the estimated 1 RM, also with 30 s of rest between sets, in the same exercises. A sham BFR protocol was applied in the ML group using the same cuff as in the LL-BFR group, but inflated to only 10 mmHg (Cerqueira and Brito Vieira, 2019).

### Blinding/masking

Participants were blinded to group allocation and were instructed not to discuss their exercise experiences with others. To ensure blinding of both participants and evaluators, all assessments and training sessions were conducted in separate locations, and exercise sessions were performed individually. To reinforce a placebo effect, participants in both groups underwent LOP assessment and were informed that BFR exercise is effective in reducing knee pain and increasing muscle strength.

### Statistical analyses

Continuous variables were expressed as the mean ± standard deviation, while categorical variables were reported as absolute and relative frequencies. Baseline sample characteristics are presented in Table 1. Data distribution normality was assessed by visual inspection of histograms and confirmed using the Shapiro–Wilk test. For the main outcome (LOP), differences between groups (LL-BFR vs. ML), time points (weeks 1, 3, 6, and 9), and group*time interactions (2 groups × 4 weeks) were analyzed using a linear mixed model, with group and time as fixed factors and baseline SBP and DBP included as random factors. When significant effects were detected, Bonferroni-corrected *post hoc* tests were applied. The same procedure was used for SBP and DBP outcomes across the intervention period. All analyses were conducted in SPSS 21.0 (IBM-SPSS Inc., Chicago, Illinois, United States), with statistical significance set at *p* ≤ 0.05.

**TABLE 1:** Sample baseline characteristics.

Variables	Group	
	ML (n = 9)	LL-BFR (n = 8)
Sex (female/male)	7/2	2/6
Knee involvement (bi/unilateral)	6/3	4/4
Age (years)	60.0 ± 7.0	59.9 ± 7.8
Height (m)	1.7 ± 0.1	1.7 ± 0.1
Body mass (kg)	72.7 ± 13.5	76.5 ± 11.3
BMI (kg/m^2^)	27.3 ± 3.9	27.0 ± 3.2
SBP (mm Hg)	120.0 ± 10.0	122.5 ± 14.9
DBP (mm Hg)	82.8 ± 4.4	82.5 ± 8.9
LOP (mm Hg)	235.0 ± 39.4	262.5 ± 37.7
LOP 60% (mm Hg)	141.0 ± 23.6	157.5 ± 22.6
Lequesne score	9.9 ± 2.4	8.4 ± 3.2
VAS (0–10 mm)	4.7 ± 1.9	3.9 ± 2.0
Estimated 1RM (hack machine)	56.1 ± 38.9	89.1 ± 51.0
Estimated 1RM (leg extension)	30.8 ± 13.7	35.8 ± 10.6

Values are presented as the means ± SD.

HI, high-load training; LL-RFS, low-load training with blood flow restriction; BMI, body mass index; SBP, systolic blood pressure; DBP, diastolic blood pressure; LOP, limb occlusion pressure; LOP60%, 60% of limb occlusion pressure; VAS, visual analogic scale.

To better approximate the interpretation of our results to clinical settings, the relative error variance (REV) was used as a cutoff value exceeding the typical measurement error of lower-limb LOP. Then, LOP values greater than 14.6 mmHg were considered clinically significant (Evin et al., 2021).

## Results

**FIGURE 2:**
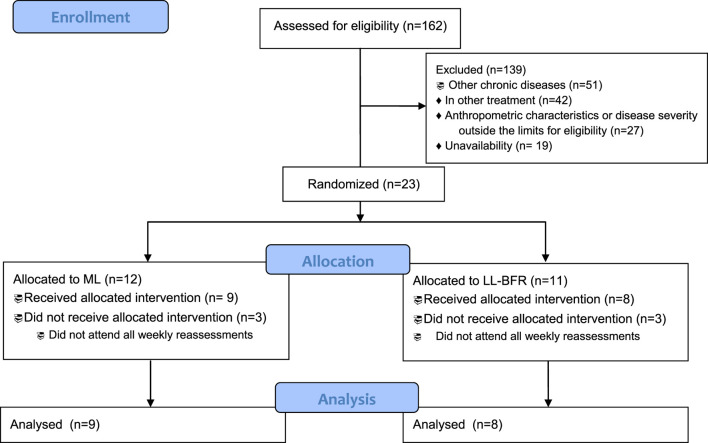
CONSORT flow diagram. LL-BFR, low-load exercise with BFR; ML: traditional moderate-load exercise.

The participant flow is presented in Figure 2. Of the 162 individuals screened, 23 met the inclusion criteria and were randomized (11 to the ML group and 12 to the LL-BFR group). During the intervention, six participants (three from each group) discontinued participation due to failure to attend all reassessment sessions. Baseline characteristics of the participants are summarized in Table 1.

**FIGURE 3:**
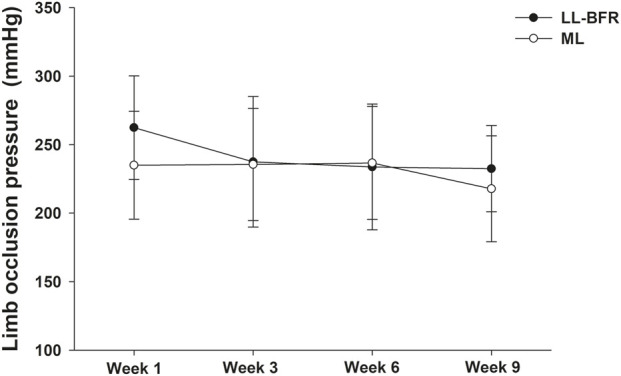
Mean ± SD of limb occlusion pressure measured at weeks 1, 3, 6, and 9 from volunteers submitted to low-load exercise with blood flow restriction (LL-BFR) or traditional moderate-load exercise (ML) intervention.

For LOP comparisons (see Figure 3), there was no significant main effect of the group (*p* > 0.05) and no significant group*time interaction (*p* > 0.05). However, a significant main effect of time was observed (*p* ≤ 0.05), indicating that LOP decreased significantly at week 9 compared with week 1, independent of the training protocol.

**FIGURE 4:**
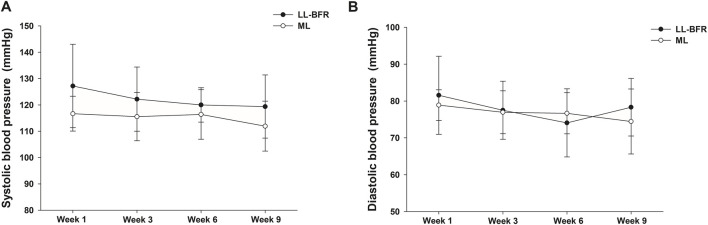
Mean ± SD of systolic **(A)** and diastolic **(B)** blood pressures measured at weeks 1, 3, 6, and 9 from volunteers submitted to low-load exercise with blood flow restriction (LL-BFR) or traditional moderate-load exercise (ML) intervention.

For SBP comparisons (Figure 4A), no significant main effects of group or time were found, nor was there a significant group*time interaction (*p* > 0.05). For DBP comparisons (Figure 4B), there was no significant main effect of group (*p* > 0.05) and no significant group*time interaction (*p* > 0.05). However, a significant main effect of time was observed (*p* ≤ 0.05), with DBP decreasing significantly at week 6 compared to week 1, regardless of the training protocol.

Table 2 presents the mean differences (95% confidence intervals) for intragroup comparisons at weeks 1, 3, 6, and 9. Considering the established criteria (REV for lower limb LOP = 14.4 mmHg), both groups exhibited a clinically significant reduction after the 9-week intervention, particularly the LL-BFR group, since the mean difference between the end and the beginning of the intervention was twice the established cutoff [week 9 minus week 1: LL-BFR, mean difference (95% CI) = −32.9 (−68.9 to 3.2); ML, mean difference (95% CI) = −17.2 (−49.0 to 14.6)].

**TABLE 2:** Mean difference (95% confidence interval) for intragroup comparisons at weeks 1, 3, 6, and 9.

Variable	Mean difference (95% confidence interval) for intragroup comparisons	
	LL-BFR	ML
LOP		
Week 9 minus week 6	−5.7 (−41.8 to 30.3)	−18.9 (−50.7 to 12.9)
Week 9 minus week 3	−10.0 (−46.0 to 26.0)	−17.8 (−49.6 to 14.0)
Week 9 minus week 1	−32.9 (−68.9 to 3.2)	−17.2 (−49.0 to 14.6)
SBP		
Week 9 minus week 6	1.4 (−6.7 to 9.5)	0.0 (−7.1 to 7.1)
Week 9 minus week 3	−3.2 (−11.3 to 4.9)	0.0 (−7.1 to 7.1)
Week 9 minus week 1	−7.5 (−15.6 to 0.6)	−1.1 (−8.2 to 6.0)
DBP		
Week 9 minus week 6	4.9 (−0.6 to 10.3)	0.0 (−4.8 to 4.8)
Week 9 minus week 3	0.2 (−5.2 to 5.7)	0.0 (−4.8 to 4.8)
Week 9 minus week 1	−3.7 (−9.2 to 1.7)	−1.9 (−6.8 to 2.9)

LOP, limb occlusion pressure; SBP, systolic blood pressure; DBP, diastolic blood pressure; LL-BFR, low-load exercise with blood flow restriction; ML, high-load exercise.

## Discussion

This study investigated potential adaptations in LOP and blood pressure and compared these outcomes between LL-BFR training and ML resistance training over a 9-week intervention in KOA patients. Although no statistically significant differences were observed between groups for LOP, SBP, or DBP, a trend toward a clinically meaningful reduction in LOP was noted, particularly following LL-BFR training.

We observed a 32.9 mmHg reduction in LOP after LL-BFR, which is twice the established cutoff for clinical significance (14.4 mmHg). In contrast, Mattocks et al. (2019) reported a non-significant mean increase of 8 mmHg in LOP following 8 weeks of LL-BFR (15% 1RM and 80% LOP, twice per week) in untrained young adults. The divergent results between our study and that of Mattocks et al. (2019), a reduction versus an increase in LOP, may be partially explained by differences in the population and the training protocol. Our participants were older, and some were hypertensive, which may have contributed to the observed reduction in LOP. We also found an ∼8 mmHg reduction in SBP after 9 weeks of LL-BFR. Moreover, Mattocks et al. (2019) implemented a higher training volume (four sets of up to 90 repetitions with weekly progression), whereas our protocol used one set of 30 and three sets of 15 repetitions with load adjustments every 3 weeks. Thus, the lower training volume in our study may also account for differences in cardiovascular adaptation (Cerqueira et al., 2021b). Clinically, the possibility of maintaining or even reducing LOP throughout a rehabilitation program is relevant because perceived effort and pain may decrease over successive training weeks (Martín-Hernández et al., 2017; Hughes et al., 2019; Teixeira et al., 2020). Conversely, unnecessary increases in LOP may heighten discomfort, impair adherence, and elevate the risk of adverse events such as venous thromboembolism, rhabdomyolysis, and bruising (Bond et al., 2019; Patterson et al., 2019; Whiteley, 2019; Spitz et al., 2020).

In the ML group, we also observed a non-significant reduction in LOP (∼17 mmHg), suggesting that LL-BFR may exert a stronger influence on lowering LOP than ML. Notably, LOP reductions occurred earlier (weeks 1–3) in the LL-BFR group and later (weeks 6–9) in the ML group. This pattern suggests earlier cardiovascular adaptation with LL-BFR. Supporting this, we found a significant main effect of time, indicating that LOP adaptations occur with strength training in both groups, although reductions were greater following LL-BFR.

With respect to blood pressure, we found no significant changes in SBP and DBP after 9 weeks. Spitz et al. (2025) similarly reported no changes in resting blood pressure in young participants following 6 weeks of LL-BFR (four sets of 2-min isometric contractions at 30% maximal voluntary contraction with 50% LOP) or ML (four maximal isometric contractions lasting 5 s). In contrast, Zhao et al. (2022) observed significant reductions in SBP after isokinetic knee extensor training in hypertensive patients aged 55–70 years: −5.2 mmHg in ML and −15 mmHg in LL-BFR, with SBP significantly lower in the LL-BFR group (mean difference: 13.2 mmHg). These stronger effects may be attributed to methodological differences: Zhao et al. (2022) implemented a longer training period (12 versus 9 weeks), greater weekly frequency (3 versus 2 sessions per week), higher training load in the LL-BFR group (30% vs. 10% 1RM), and higher cuff pressure (130% SBP vs. 60% LOP). Regarding DBP, our findings are consistent with those by Zhao et al. (2022), showing small, non-significant reductions in both groups.

Our findings should be interpreted in light of several limitations. First, the sample size was small, and data variability was high. Second, the training protocol used very low loads (10% 1RM) and moderate occlusion pressure (60% LOP). Pressures less than 70% LOP may not significantly reduce arterial blood flow (Souza et al., 2025), and higher occlusion pressures (80% LOP) may be necessary to elicit neuromuscular adaptations when exercising loads < 20% 1RM (Lixandrão et al., 2015). Thus, stronger LOP adaptations might require higher cuff pressures when very low loads are prescribed. We also assessed LOP in supine, which may have underestimated occlusion pressures during exercise. Previous research (Kamiş et al., 2024) has shown that the position used for LOP assessment can influence performance and perceptual responses during LL-BFR exercise and may also affect cardiovascular responses. Additionally, we did not directly evaluate vascular outcomes (e.g., endothelial function, arterial compliance, arterial stiffness, and arterial diameter), which may influence LOP (Cerqueira et al., 2021b). Finally, although Mattocks et al. (2019) observed an increase of 9 mmHg in LOP following 8 weeks of upper limb LL-BFR (15% 1RM; 80% LOP), our trial involved lower limbs, underscoring the need for future studies to investigate whether adaptations differ by training site and clinical population.

## Conclusion and future perspectives

Our results indicate a non-significant trend toward a reduction in LOP following 9 weeks of LL-BFR in patients with KOA, with a mean point estimate exceeding a pre-defined threshold for clinical significance, although the wide confidence intervals indicate substantial uncertainty. Furthermore, SBP and DBP showed no significant changes, and no group differences emerged across outcomes. To our knowledge, this is the first study to evaluate adaptations in LOP and blood pressure in clinical KOA patients. From a practical perspective, these findings suggest that LOP can be maintained—or even reduced—throughout a rehabilitation program, potentially making the application of LL-BFR in clinical practice less burdensome. Future studies should investigate LL-BFR protocols with periodic adjustments in occlusion pressure, longer intervention durations, and training loads greater than 10% 1RM to better determine the impact of these variables on LOP in clinical populations.

## Data Availability

The raw data supporting the conclusions of this article will be made available by the authors, without undue reservation.
